# Does sepsis cause increased long-term mortality? a systematic review

**DOI:** 10.1186/2197-425X-3-S1-A759

**Published:** 2015-10-01

**Authors:** M Ambler, V Mahalingasivam, A Jones, K Rowan, GD Rubenfeld, M Shankar-Hari

**Affiliations:** Guy's and St Thomas' NHS Foundation Trust, Critical Care Medicine, London, United Kingdom; Intensive Care National Audit and Research Centre, London, United Kingdom; Sunnybrook Health Sciences Centre, Interdepartmental Division of Critical Care Medicine, Toronto, Canada

## Introduction

Acute mortality for adult critically ill patients with sepsis is improving. There is ample evidence that survivors of sepsis have an ongoing risk of death that extends beyond the acute illness.[1] However, whether sepsis is an independent and potentially causal factor in these outcomes is unclear. Since sepsis likely increases acute mortality compared with other conditions, studies to address this question would have to separate acute from post-acute mortality, adjust for confounding variables, and identify appropriate control populations. We performed a systematic review of studies reporting longer-term mortality following sepsis in adult ICU patients to specifically identify those that contained sufficient analyses to address issues of causality.

## Objectives

Our objective was to evaluate whether studies reporting longer-term outcomes have the design features and/or statistical analyses to support causal inferences about the association between sepsis and longer-term mortality.

## Methods

A systematic search for non-randomized and randomized clinical studies in the Medline (1946-2013) and Embase (1974-2013) databases was performed (Ovid platform). Search terms included the following 'mp' terms, MESH headings and combinations thereof- sepsis, septic shock, septi?aemia, outcome, quality of life, cohort studies, and randomi?ed controlled trials. Results presented as median [IQR] of proportions. Acute mortality refers to ICU/hospital/up to 30-day and [1-acute mortality] was defined as acute illness survival. Post-acute mortality is the proportion of deaths in acute illness survivors over the long term follow up period.

## Results

The search identified 4034 articles, excluding duplicates, and 23 studies reported one-year or longer-term, post-acute mortality. In these studies, 65.1% [53.1%-72.1%] sepsis patients survived the acute illness. The one-year, post-acute mortality in sepsis survivors was 24.4% [18.4% - 42.8%]. Only four studies reported a non-sepsis control arm and their design/analysis features precluded causal inferences. [2–5]

## Conclusions

Based on our systematic review, there is limited evidence to support the hypothesis that sepsis causes an increase in post-acute mortality. Future studies reporting acute and post-acute mortality, adjusted for confounding, should incorporate multiple control populations to help us answer this question.

## Grant Acknowledgment

NIHR UK, Biomedical Research Centre, Kings Health Partners

**Table 1:** Table 1

Author [time]	Sepsis subtype	Acute Mortality Sepsis	Post-Acute Mortality Sepsis	Description of Controls	Acute Mortality Controls	Post-Acute Mortality Controls	Analysis feature for causality
Ghelani D et al [5 years]	(a) ICU Admission (b) Nosocomial	(a) 41.9% (b) 64.5%	(a) 26.5% (b) 15.1%	(a) Non sepsis ICU (b) Infected Hospital (c) Non infected Hospital	(a) 25.5% (b) 9.5% (c) 2.5%	(a) 31.5% (b) 28.6% (c) 18.8%	Multivariable adjustment
Korosec-Jagodic H et al [2 years]	Sepsis	58.0%	11.0%	Trauma	38.0%	5.0%	-
Quartin AA et al [1 year]	(a) Uncomplicated Sepsis (b) Severe sepsis (c) Septic shock	(a) 23.0% (b) 47.0% (c) 57.0%	(a) 23.0% (b) 24.0% (c) 23.0%	3 risk adjusted hospital controls for each sepsis subtype	(a) 8.0% (b) 10.0% (c) 9.0%	(a) 16.0% (b) 18.0% (c) 17.0%	Multivariable adjustment
Regazzoni CJ et al [1 year]	Severe sepsis	21.8%	37.2%	Non-septic patients	14.7%	20.3%	Multivariable adjustment
